# Supplementary material for: From effective biocontrol agent to successful invader: the harlequin ladybird (Harmonia axyridis) as an example of good ideas that could go wrong
Source: PeerJ. 2017 May 16;5:e3296. doi: 10.7717/peerj.3296 (PMC5436555; doi:10.7717/peerj.3296)
Supplement: Supplemental Information 2 [file peerj-05-3296-s002.docx]

**Survey Template**

Subject: Invasive ladybird: worldwide survey

Dear [insert name],

As a scientist who have studied the *Coccinellidae* family, we are hoping you might be able to help us with our work on invasive ladybirds by answering 6 quick questions, which should not take more than 5 minutes.

Specifically, we are keen to document the distribution and origin of the invasive harlequin ladybird (*Harmonia axyridis*) worldwide.

We hope to construct a comprehensive database combining information from around the world to enhance our understanding of invasive ladybirds, and invasive species in general.

We would be extremely grateful if you could spare a few minutes to answer the following questions; we attach as well a word file with them, in case you find it easier to answer there.

1. Are you aware of the presence of harlequin ladybirds within the cities and towns, crop field areas or reserves in [insert country/region]? Are they considered native or invasive?(If not, please proceed to question 6). If possible, please specify regions or particular locations.
2. If you have seen them, are the harlequin ladybirds found throughout the region, or in localised parts only?
3. Do you know anything about the origin of the introduction(s)? For example, when, where and/or why they were introduced?
4. Are you aware of any negative effects on habitats, ecosystems or other native species in areas where the ladybirds are found (e.g. predation on/competition)?
5. Are you aware if any native species have gone extinct due to the presence of the harlequin ladybird? If so, please specify which ones.
6. Can you think of any other information regarding harlequin ladybirds in your region that might be of interest to us?

Our goal is to produce an online map showing the contemporary distribution of the harlequin ladybird and we will of course acknowledge your help in contributing records (including negative ones) to it.

Many thanks for your help,

With best wishes,

Dr. Mora Camacho-Cervantes and Dr. Ek del Val de Gortari

Lab. Interacciones bióticas en hábitats alterados

Universidad Nacional Autónoma de México, Campus Morelia.

http://ww2.oikos.unam.mx/CIEco/interacciones/
